# Medical Society of Berlin

**Published:** 1889-05

**Authors:** 


### MEDICAL SOCIETY OF BERLIN.

### Prophylaxis of Tuberculosis.

This subject was presented by Dr. Cornet. Cornet was specially qualified for an address on such a subject. He has for the last two years been studying experimentally in Professor R. Koch's Hygienic Institute, with a view of determining the behavior of the tubercle bacillus both within and without the body. The speaker discussed the doctrine that led to the false conclusion that certain people had an immunity against it. It was shown that the air was the chief carrier of the virus. All tubercle bacilli, however, had their origin in the bodies of animals or men, and here was the starting point of the prophylaxis of phthisis, and as the bacillus could not be destroyed within the body the aim should be to render it innocuous the moment it left it. Fæces and urine of phthsical patients sometimes contained bacilli, but no danger arose from them, as the rapidly growing bacteria of decomposition quickly destroyed them. An infection of the intestinal tract could, however, be caused by the milk or flesh of tuberculous animals as was shown in the case of children. The great source of the infection, however, was the phthsical individual himself when the affection was in the lungs. Here also there was an incorrect notion that the virus was contained in the breath itself. He believes that such is not the case, for, according to the investigations of Nägeli, tubercle bacilli are never found in the expired breath of phthisical patients. The danger lies in the sputum alone, and then only when it becomes dried, pulverized, and caught up as dust into the air. Sputum is not easily pulverized, however, and moreover there are protective arrangements for preventing the ingress of such virus in the nasal passages and the respiratory tracts, where the bacilli are caught and sent back to the outer air by the ciliated epithelium of the air passages. The sputum is most dangerous when expectorated on to the ground, or into a handkerchief, as then the conversion of it into dust is most favored. In his numerous investigations in hospitals and private dwellings where the sputum was ejected on to the floor or into handkerchiefs, he always found virulent bacilli in the air or on the walls whilst they were constantly absent where expectorations took place into proper vessels. It was no wonder, therefore, that those belonging to consumptive patients, and especially the children of consumptive mothers, suffered so much from the disease. As regards prophylaxis, it is first of all requisite that, whether tuberculous or not, every one expectorating in a closed space should do so in a proper vessel, and phthisical patients should be taught always to expectorate in a vessel, never on the floor or in a handkerchief. Spitting-cups might be quite safely emptied into the closets, and they only require a little water at the bottom to prevent drying. In case of death the walls of the chamber should be rubbed down with freshly-baked bread, the floor washed, the bed and clothing disinfected in super-heated steam. Generally speaking it was advisable to rub down the walls with bread on any change of residence. Consumptive patients should be separated from others in general hospitals. By following out the principles of prophylaxis he had mentioned, he expected considerable diminution in the ravages from that disease.

In the discussion that followed, Dr. B. Frankel pointed out that some portion of the sputum containing bacilli would often be retained in the saliva of a phthisical patient; that was an old observation. He related the case of a healthy young clerk being engaged in an office with a consumptive principal. Both worked at the same desk, and both were in the habit of nibbling their penholders. After a time the clerk took ill of tuberculosis and died, after he had in his turn infected his healthy mother.

Dr. Cornet pointed out, in reply, from statistics compiled by himself, that 63 per cent. of nurses fall victims to tuberculosis. A healthy girl devoting herself to this calling died 21 years before the other population, and this difference in the mortality was entirely due to the increased amount of phthisis. He remarked further that in orphanages where strict prophylaxis was followed, tuberculosis was kept away, although the children might be the offspring of phthisical parents. In the orphanage at Nürnberg, where there were 400 children, mostly of tuberculous parentage, only two or three died of phthisis in the course of eight years. In hospitals the time of greatest danger was that of dusting and cleaning up.

